# Supplementary material for: Are carbon emissions trading and green financial instruments synergistic? -Comprehensive quantitative research based on content analysis
Source: PLoS One. 2024 Mar 7;19(3):e0298601. doi: 10.1371/journal.pone.0298601 (PMC10919601; doi:10.1371/journal.pone.0298601)
Supplement: S1 Data — (ZIP) [file pone.0298601.s001.zip › Data/地级市Z_F工作报告.zip!/╣π╬≈/╬α╓▌╩╨/└┤╫╘╣1⁄2╓┌║┼í╛┬φ┐╦╩2╛▌═°í┐_2008.docx]

各位代表：

　　现在，我代表市人民政府向大会作工作报告，请予审议，并请市政协委员和其他列席会议的同志提出意见。

　　2007年工作回顾

　　2007年，我市在自治区党委、政府和市委的正确领导下，在市人大和市政协的监督支持下，坚持以邓小平理论和“三个代表”重要思想为指导，全面贯彻落实科学发展观，围绕市十二届人大二次会议确定的各项奋斗目标，深入实施开放带动和项目带动两大战略，经济社会出现了多年来少有的快速发展势头。全市实现生产总值326.76亿元，比上年增长15.7%；财政收入27.03亿元，增长17.1%；全社会固定资产投资完成151.37亿元，增长24.1%；万元地区生产总值能耗和万元规模以上工业增加值能耗分别下降3%和4.2%；社会消费品零售总额111.27亿元，增长13.5%；外贸出口总额3.22亿美元，增长10.7%；城镇居民人均可支配收入11362元，增长20.2%；农民人均纯收入3272元，增长14.5%。

　　工业经济进一步壮大　全市规模以上工业完成总产值269.48亿元，增长53.8%；规模以上工业企业盈亏相抵后实现利润8.93亿元，增长83.44%。全市年产值超亿元的企业62家，比上年增加33家；重点工业企业完成产值50.45亿元，占全市规模以上工业总产值的18.72%。工业对经济增长的贡献率达64.6%，年纳税超1000万元的企业24家，年纳税10万元以上的企业1205家。

　　农业基础地位进一步加强　全年农林牧渔业总产值完成102.8亿元，增长4.3%。投资1000万元年产1000吨成品六堡茶项目和投资1.1亿元的同舟农产品物流配送中心项目已启动建设。全市完成病险水库除险加固11座，建成饮水安全工程35项，解决了5.25万人的饮水困难问题，农村生产生活条件进一步改善，社会主义新农村建设取得新成效。

　　重大项目建设进一步加快　全年完成投资88亿元，完成计划的80.1%。新开工重点项目75个，完成计划的82.4%。列入自治区统筹推进和重点监控的11个项目全部开工建设。长洲水利枢纽已有3台机组并网发电，林浆纸一体化项目部分评估报告已上报国家有关部门核准；木薯燃料乙醇项目前期工作推进机构已选定，梧州进口再生资源加工园区项目前期工作加快推进。

　　承接产业转移取得新成效　全市实际利用境外资金1.38亿美元，增长6.3%；引进市外境内资金86.94亿元，增长17.1%。新引进产业转移项目231个，投资总额76.26亿元，到位资金26.5亿元。工业园区和集中区完成基础设施建设投资4.18亿元，完成工业总产值64.8亿元。全市初步形成了“两园六区”的产业集聚格局。成功举办了第四届梧州国际宝石节，签定项目34个，合同投资总额28.33亿元。

　　立体交通格局初步形成　全年完成交通固定资产投资27.67亿元，是我市交通建设史上投资最大、项目最多、效果最好的一年。岑梧高速公路建成通车，马梧高速公路建设加快，洛湛铁路梧州段进展顺利，与广西机场集团公司合作开通了梧州至南宁、桂林、西安等多个城市的航线，西江Ⅱ级航道梧州段整治以及一批县际二级公路和乡村公路建设顺利推进。

　　城市建设再上新台阶　全年完成城市基础设施建设投资10.27亿元，一批城市标志性工程建设顺利推进；廉租房和经济适用房建设步伐加快；全市城镇化水平达33%，比上年提高0.4个百分点。骑楼城—龙母庙景区被评为国家4A级旅游景区；全年接待旅游人数、旅游总收入分别比上年增长16.5%、18.4%。市区荣获第六届全区市容“南珠杯”竞赛奖。

　　财政金融支撑作用加强　市本级财政安排3000万元用于工业园区建设和支持重点企业发展；全年财政涉农补贴比上年增长24%，扶持教育、公共医疗卫生和社会保障事业发展资金分别比上年增长20.5%、43.6%和9.6%。金融机构全年发放贷款96.11亿元，比上年增长29.5%；各项贷款余额比年初增加27.7亿元，增长22.5%，全年信贷投放量和增速创历史新高，为全市经济发展提供了有力的资金支持。

　　节能减排任务全面完成　我市列入自治区监控的15家重点耗能企业全年完成节能量7.33万吨标准煤，完成任务的530%；实施节能改造项目5个，节约能源2.58万吨标准煤。全市关停小土纸作坊380家、淘汰5家钢铁企业落后生产设备以及一批工艺落后的砖瓦窑，关停了2家火电厂。我市确定的35家重点污染源企业废水治理设施正常运行率保持在96%以上。全年化学需氧量排放量比上年削减8.86%，二氧化硫排放量削减1.98%，全面完成了自治区下达的目标任务。

　　民生工程迈出新步伐　全市新增就业1.59万人，城镇登记失业率3.95%，低于控制数0.55个百分点。全市养老、医疗、失业、工伤和生育保险参保人数稳步增长，各项保险基金按时足额发放。城乡居民最低生活保障制度进一步落实，启动了城镇居民基本医疗保险和市级失业保险统筹试点工作，率先在全区把在校中小学生纳入城镇居民基本医疗保险。城市社区卫生服务覆盖率达到90%以上，新型农村合作医疗参合率达到76.7%。年初确定为民办的10件实事全部完成，解决了一批关系群众切身利益的现实问题，特别是地质灾害整治取得了阶段性成果。

　　各项社会事业全面发展　积极实施并圆满完成第三轮科技创新计划。“两基”工作通过了国家验收；梧州职业学院将于今年秋季实现招生。公共卫生和基本医疗服务体系不断完善。文化事业和文化产业加快发展，文艺精品创作取得新突破。广播电视覆盖率进一步提高，骑楼城影视基地影响逐步扩大。竞技体育和全民健身活动蓬勃发展。质量监督和食品药品安全专项整治活动及食品放心工程取得好成绩。人口和计划生育工作稳步推进，初步扭转了被动落后的局面。深入实施人才强市战略，人才队伍进一步发展壮大。安全生产和社会稳定工作得到强化，我市被命名为2005—2007年自治区平安市。国防动员建设取得新成效，军政军民关系融洽，第三次荣获“全国双拥模范城”称号。物价、审计、统计、扶贫开发、农机、气象、地震、人防、民族、宗教、对台、侨务、新闻出版、史志、档案、工青妇、老龄、残疾人等各项工作都取得了新成绩。

　　各位代表，回顾过去一年的政府工作，我们深感这些成绩来之不易，这是自治区党委、政府和市委正确领导的结果，是在历届政府打下的良好基础上，全市上下抢抓机遇、开拓进取、艰苦创业、团结拼搏的结果。在此，我代表市人民政府，向在各个领域和岗位上辛勤劳动、作出贡献的全市人民，向解放军和武警驻梧部队、政法干警，向各民主党派、工商联、无党派人士和各界朋友，向中直、区直驻梧单位，表示衷心的感谢并致以崇高的敬意！

　　在肯定成绩的同时，我们也清醒地看到，我市经济社会发展仍然存在不少问题，主要是经济总量不大，支撑经济持续快速发展的重大项目特别是工业项目较少；财政收支矛盾比较突出，产业结构不够合理；农民持续增收难度大；节能减排压力较大；部分低收入群众生活比较困难；政府自身建设有待进一步加强。对于这些问题，我们将高度重视，采取有效措施认真解决。

　　2008年工作总体要求和目标任务

　　今年是全面贯彻落实党的十七大作出战略部署的第一年，是实施“十一五”规划承上启下的一年，也是改革开放30周年和自治区成立50周年，做好今年的经济社会发展工作意义十分重大。根据市委提出“两年打基础，三年大发展”的部署，今年政府工作的总体要求是：以党的十七大精神为指导，坚持科学发展、加快发展、和谐发展，坚持解放思想，继续实施开放带动和项目带动两大战略，加快推进工业化、城镇化和农业产业化，加强区域经济合作，切实抓好节能减排，全力把我市建设成为承接产业转移示范基地，区域性加工制造、物流、商贸和旅游休闲基地，连接泛珠三角经济圈和泛北部湾经济圈的区域性枢纽城市。

　　今年我市经济社会发展的主要目标建议为：地区生产总值增长13%，力争15%；规模以上工业增加值增长26%，力争31%；万元地区生产总值能耗和万元规模以上工业增加值能耗分别下降1.7%和3.2%；主要污染物排放总量控制在自治区下达的指标以内；全社会固定资产投资增长20%，力争22%；财政收入增长13%，力争15%；外贸出口总额增长15%，实际利用外资增长5%，引进市外境内资金增长30%；社会消费品零售总额增长15%；城镇居民人均可支配收入增长10%；农民人均纯收入增长8%；城镇登记失业率控制在4.5%以内；人口自然增长率控制在10‰以内。

　　面对加快发展的历史重任，我们必须进一步解放思想，牢固树立忧患意识、责任意识、发展意识，勇于变革，勇于创新，永不僵化，永不停滞；必须营造浓厚的干事创业氛围，让想干事的人有机会，能干事的人有舞台，干成事的人有位置；必须大力弘扬求真务实的精神，真抓实干，察实情、讲实话、出实招、办实事；必须高度关注民生，保障民生，不断改善民生；必须调动一切可以调动的积极性，激发全民创业的热情。为此，今年具体抓好以下10项工作：

　　一、举全市之力加快工业发展，扎实推进“五大”建设

　　加强现有工业企业建设　继续落实好扶持工业企业政策和15条保护措施，重点扶持30家有效益、有市场、有前景的企业，力争今年30家重点企业实现税收占市本级税收的40%以上。抓好企业技术改造和优化升级，加快建立以企业为主体、以市场为导向、产学研相结合的技术创新体系，增强企业竞争力。突出抓好企业异地搬迁和扩大再生产，力争全市技术改造投资增长20%以上。加快推进蛋白肠衣、奥奇丽、华虹丝绸、日成林化等条件成熟企业上市的前期工作，力争今年实现上市2家以上。大力引进有实力的担保公司，解决我市中小企业融资难、贷款难问题。

　　加快新增工业项目建设　主动适应国家宏观调控政策，努力筹措建设资金，组织推进100个投资3000万元以上的工业重点项目。加强新建工业项目服务，切实解决项目在土地、资金、环保等方面的问题，确保工业项目建设取得新突破。

　　加快工业园区和集中区建设　梧州工业园区要完成融资1亿元，力争达到2亿元，新建工业厂房30万平方米以上，新增建设用地800亩以上；苍梧、岑溪、藤县分别新建工业厂房15万平方米以上，蒙山、万秀、蝶山、长洲分别新建工业厂房4万、7万、6万、8万平方米以上，梧州进口再生资源加工园区新建工业厂房8万平方米以上，确保全市新增工业厂房面积108万平方米以上。加快组建园区、集中区开发投资公司。完善工业园区规划，争取3个城区和蒙山县工业集中区年内通过自治区A类产业园区确认。制定完善园区经济目标管理考核制度，培育园区、各集中区骨干企业，形成特色的产业集聚区，把我市打造成为区域性加工制造基地。全市园区、集中区力争完成固定资产投资20亿元，完成工业增加值21亿元，完成税收3亿元。

　　全力推进重大工业项目建设　今年安排重点工业项目10个，总投资148亿元，年内计划完成投资8亿元。积极推进林浆纸一体化、木薯燃料乙醇项目前期工作，力争年内开工建设；抓好进口再生资源加工园区的基础设施建设，加快三威林产、新华电池、森川松脂、天鹅蓄电池、双钱实业易地搬迁和技改扩产步伐；促进制药集团新药产业化和六堡茶加工生产项目尽快建成投产，发挥效益。

　　大力加快县域工业建设　立足县域资源特点，以民营经济为主体，以承接产业转移为主要途径，积极发展农副产品精深加工、矿产品加工、水电等特色工业，发展中小企业，培育支柱产业。完善工业集中区服务功能，促进工业企业集聚发展，推动县域经济发展全面提速，努力实现县域工业产生的税收占全市工业税收的50%以上。

　　二、加快新农村建设步伐，扎实推进“五大”惠农工程

　　农民增收工程　切实稳定粮食种植面积，确保粮食总产量稳定在90万吨以上。继续抓好砂糖橘、六堡茶、木薯、速丰林、三黄鸡、瘦肉型猪等十大农业优势产业发展，推进农业优势产业标准化生产、基地化建设。积极推进科技特派员“创业年”活动。扶持发展农产品加工业，培育龙头企业和合作经济组织，实现工农结合互动，带动农业产业化发展，促进农产品转化增值和农民增收。

　　农村水利工程　抓好农村水利设施建设，加大病险水库除险加固力度，增加有效灌溉面积。加强农村饮水安全工程建设，进一步解决农村饮水安全问题。加快藤县防洪堤工程建设，抓紧长洲防洪堤正阳堤段扩建应急工程建设，加快推进长洲岛防洪堤工程前期工作。

　　农村通达工程　加快乡村公路建设，努力实现村村通公路。加强农村电网、农业技术推广网、农机服务网、农资配送网等建设，建立完善农业社会化服务体系。

　　农民健康工程 加快推进新型农村合作医疗制度，提高农民参合率。加大城市对口支援力度，分批轮训农村卫生技术人员，提升乡村卫生服务水平，努力实现农民“小病不出村，大病能及时救治”。大力开展农村沼气池建设，配套搞好改水改厕工作。

　　农民培训工程　加大对农民工和农村新生劳动力培训力度，提高农民综合素质，加快农村富余劳动力转移步伐，拓宽农民增收渠道。继续做好新农村建设指导员选派工作，全面推进社会主义新农村建设。

　　三、夯实发展基础，努力建设区域性物流、商贸、旅游休闲基地

　　继续完善立体交通体系　加快梧州至马江、岑溪至兴业、岑溪至筋竹高速公路建设进度，建成藤县至容县、藤县潭东至太平、蒙山黄村至昭平二级公路，积极推进藤县至苍梧一级公路、市区至藤县津北公路和梧州至平南、岑溪至水汶高速公路的前期工作。加快推进洛湛铁路梧州段、岑溪至罗定地方铁路建设；配合推进南宁至广州铁路，力争今年开工建设。做好港口、码头建设规划，配合做好贵港至梧州Ⅱ级航道整治，开展桂江航道整治工程的前期工作；争取梧州港赤水作业区码头开工建设，加快河西码头新址工程建设，完成河西码头搬迁。积极争取开通梧州至珠海等空中航线。

　　努力打造区域性物流基地　做好航空物流园区、公路物流园区、铁路物流园区和港口物流园区的规划，逐步完善基础设施，使我市成为桂东南区域性的物资运输、配送、中转、仓储、加工和信息处理中心。大力发展和培育现代物流企业，推进同舟农产品物流、易达物流、宝石物流、烟草物流、万秀区医药物流中心建设及基础设施配套建设。积极引进国内外知名物流企业参与我市物流业建设和经营，不断提高物流企业的服务水平和辐射能力。

　　努力打造区域性商贸基地　抓好全市商业网点规划的编制工作，优化商业网点布局，围绕建设区域性商贸中心，培育以两广批发市场为核心的大型专业市场。继续组织实施 “双百市场工程”和“万村千乡市场工程”建设。加快发展现代服务业，增强消费对经济增长的拉动。切实加强城乡市场监管，进一步整顿和规范市场经济秩序。

　　努力打造区域性旅游休闲基地　整合白云山景区旅游资源，推进四恩寺整体工程建设和配套项目建设，加快西江明珠广场和云山海泰项目建设，为白云山景区创建国家4A级旅游景区奠定基础。抓住骑楼城—龙母庙景区获得国家4A级旅游景区的机遇，打造好龙母民俗文化旅游。进一步挖掘中山纪念堂、李济深故居、太平天国封王遗址、袁崇焕故里等历史文化古迹资源，丰富旅游业内涵。继续抓好各县（市）旅游景区、景点和长洲水利枢纽库区水上娱乐、休闲度假区的规划建设。加强与广东“广之旅”和南湖国际旅游集团的合作，打造“广肇梧”两广省界游和桂东宗教文化游等黄金旅游线路。

　　四、加快城市建设步伐，扎实推进城镇化进程

　　进一步拓展城市发展空间　以《城乡规划法》颁布实施为契机，充分发挥城乡规划的龙头作用，统筹城乡发展。按照构建区域性枢纽城市的要求，重点推进红岭片开发建设。认真做好龙圩、塘步、埌南、新地环城工业经济圈和苍梧至藤县、苍梧至岑溪公路沿线城镇经济带的规划建设。结合地质灾害治理，加快旧城改造步伐，重点抓好白垢村、河东汽车站地块、华杰同富购物中心项目改造建设。

　　加快城市基础设施建设步伐　加快西江三桥、火车站站前大道、三龙水厂配套工程等项目建设。继续推进一批城市标志性工程建设和市体育中心等社会公益性项目建设。继续抓好城市街道改造，进一步解决小街小巷路不平、灯不亮、渠不通的问题。加快县城和重点镇建设，着重解决基础设施不配套的问题。

　　继续开展“城乡清洁工程”　坚持深入持久地开展“城乡清洁工程”，加大城乡市容环境综合整治工作力度，强化严管重罚措施，完善城镇市容环境管理体制，发挥街道、社区、居委会和机关单位、居民参与城市管理的作用，不断改善城乡人居环境和发展投资环境，提高城市品位。

　　五、加大对外开放力度，大力承接产业转移

　　接轨珠三角融入北部湾，积极参与多区域合作　抓住多区域合作的新兴机遇，主动接轨珠三角，融入北部湾，积极参与国内外区域经济合作以及“百企入桂”等重大活动，加强我市与东盟国家、粤港澳和长三角地区省市的合作，努力拓展合作空间，不断扩大合作成果。全力办好第五届梧州国际宝石节，争取一年上一个新台阶。

　　坚持有效招商方式，提高引资质量　坚持以珠三角地区为招商引资主战场，创新招商方式，强化目标考核，推进选择性招商。各级政府主要领导要亲自抓项目，8个招商小组要继续发挥作用，坚持责任不变、任务不变、措施不变、目标不变，完善激励问责机制，确保取得良好成效。做好中国—东盟博览会、泛珠三角经济合作、宝石节和各类经贸洽谈会已签约项目的跟踪落实，提高合同履约率、项目开工率和资金到位率。

　　突出地方特色，努力扩大外贸出口　突出抓好日用轻工、电子、林化、化工等行业龙头企业的培育，扩大电池、电子、松香、机械、钛白粉、人工宝石等传统优势产品出口，力争今年市属外贸出口总额达3.25亿美元，增长15%。加强口岸工作，进一步完善“大通关”的工作机制，提高通关效率，促进出口增长。

　　六、抓好节能减排工作，加快推进生态文明

　　抓好节能减排目标考核　认真落实节能减排实施方案，深入开展“全民节能减排行动”，把节能减排指标完成情况作为各县（市、区）、各部门行政领导和领导班子年度政绩考核的重要内容，实行严格的问责制和“一票否决制”。坚决淘汰一批高能耗高污染的“五小”落后生产设备，为优势产业、优势项目腾出更多的能耗空间和环境容量。完善政策措施，建立健全节能减排激励机制、约束机制和淘汰落后产能退出保障及补偿机制。今年市本级财政安排节能减排专项资金2000万元，各县（市、区）也要安排100至200万元节能减排专项资金。

　　抓好重点企业节能　继续抓好自治区重点监控年耗1万吨以上标准煤企业的节能，加强重点耗能企业监管，确保完成全年节约标准煤的任务。组织重点耗能企业实施节能降耗技术改造，大力推广和使用节能先进技术。大力发展循环经济，在建设资源节约型、环境友好型社会上取得成效。

　　抓好污水处理厂建设　加快市第一污水处理厂建设步伐，力争今年4月份建成投产；争取今年上半年开工建设市第二污水处理厂、梧州工业园区和各县（市）污水处理厂。

　　七、加强财税工作，促进财政金融平稳运行

　　加强财源建设，做大财政“蛋糕”　大力发展工业，稳定支柱产业、支柱财源，做大经济总量和财政收入总量，促进财政收入再上新台阶，力争今年突破31亿元。积极扩大中小税源，努力实现年纳税10万元以上企业1300家。强化税收征管，堵塞税收漏洞，做到应收尽收。加大非税收入的征管力度，严格按照“收支两条线”的要求和部门综合预算的规定，有序地把非税收入纳入预算管理，统筹安排使用。

　　加强财政管理，优化支出结构　今年安排1亿元资金全力支持工业发展，支持企业技改和做大做强。积极筹集建设资金，重点支持科技、教育、文化、卫生等事业和公共设施建设，保障人民群众身体健康，提高抗灾防灾能力和应对公共突发事件能力。以确保重点支出为核心，优化支出结构，进一步落实“两免一补”、就业和社会保障等各项民心工程，切实保证各项法定支出、工资发放和党政机关正常运转。继续深化完善财政投资评审、政府采购、国库集中支付等改革，推进财政资金绩效评价。

　　加强金融生态环境建设，营造良好投融资环境　进一步完善政银企的沟通协调机制，积极创造条件，拓宽融资渠道，着力解决项目优良、第一还款来源明确稳定、担保载体有效的企业贷款问题，切实为项目发展融资提供保障。大力发展地方金融，支持金融改革，加强金融监管和金融服务。规范社会信用秩序，努力培育良好的信用环境，发挥金融保险对地方经济和社会发展的推动作用。

　　八、着力改善民生，扎实推进和谐社会建设

　　加强就业和社会保障工作　全市城镇新增就业人数1.2万人以上，城镇登记失业率控制在4.5%以内。在扩大就业总量的同时，通过规范劳动关系，严格落实最低工资标准。继续完善城镇职工基本养老、基本医疗、失业、工伤和生育保险制度，扎实推进失业保险市级统筹试点工作，建立健全失业保险预警机制。全面推进新型农村合作医疗和城镇居民基本医疗保险试点，逐步实现“全民医保”。完善城乡居民最低生活保障制度，确保应保尽保。争取全市城镇企业职工参加基本养老保险人数13万人，失业保险参保人数10.9万人，基本医疗保险参保人数21.2万人，工伤保险参保人数9.6万人，生育保险参保人数8.4万人。

　　推进扶贫开发　抓好第二批27个贫困村的整村推进扶贫开发。动员社会力量支持参与扶贫开发。力争今年第二批贫困村90%以上的人口稳定解决温饱问题。加大库区移民工作力度，进一步改善库区移民生产生活条件。

　　加强防灾减灾基础建设　继续多渠道筹集资金，加快推进地质灾害治理、城市防洪体系建设。建立健全自然灾害、事故灾难、公共卫生和公共安全等监测预警体系和信息报告、应急救援、社会动员机制。提高全民防灾抗灾意识及应对、处置公共安全、突发性事件和防灾救灾能力。

　　九、坚持为民办实事，让群众共享更多发展成果

　　今年是自治区成立50周年大庆，我们要以此为契机，集中力量为民办好以下12件实事：

　　（一）实施城乡医疗惠民工程。一是推进和健全新型农村合作医疗制度。筹措资金1.8亿元，使全市农民参合人数达到200万人，参合率达到80%以上；二是推进城镇居民基本医疗保险试点。筹措资金500万元，其中市本级财政筹措320万元，按每人每年不低于30元标准补助，力争参保人数达16万人，参保率达60%以上；三是全面建立城乡医疗救助制度。筹措资金137万元，保障参加城镇居民基本医疗保险的低保对象按每人每年不低于60元标准补助，参加新农合的农村五保户和低保户按每人每年10元标准补助。

　　（二）实施农村人口饮水安全工程。筹措资金3227万元，解决7.5万农村人口饮水安全问题。

　　（三）实施教育惠民工程。一是筹措资金1988万元，用于职业教育攻坚；二是筹措资金30万元，继续资助考上大学的贫困家庭子女；三是对农村义务教育阶段经济困难寄宿生补助生活费；四是免除城市义务教育阶段学生学杂费。

　　（四）实施城乡最低生活保障工程。全面提高城乡最低生活保障标准，城市最低生活保障标准由每人每月195元提高到210元，农村最低生活保障标准由每人每月15元提高到20元。

　　（五）实施廉租房和经济适用房建设工程。筹措资金5400万元，确保廉租房竣工面积1.6万平方米，经济适用房竣工面积4万平方米。

　　（六）实施农村公路建设工程。筹措资金6204万元，建设农村公路35条，建设总里程190.6公里，新增40个行政村通硬化公路。

　　（七）实施沼气池建设工程。筹措资金1556万元，新建沼气池8190座。其中非贫困村沼气池6490座，贫困村沼气池1700座。

　　（八）实施管道燃气进小区工程。筹措资金1750万元，基本完成河西管道燃气改造工程，满足8800户居民使用需要。

　　（九）实施市区地质灾害整治工程。筹措资金5000万元，完成市八中边坡、意园冲、珠山、榜山等地质灾害隐患整治。

　　（十）实施广播电视无线城乡覆盖工程。筹措资金384万元，建设县（市）广播电视转播台，扩大中央、广西和本地广播电视节目的有效覆盖。

　　（十一）实施农村贫困残疾人危房改造工程。筹措资金250万元，对全市500户农村贫困残疾人的住房进行改造。

　　（十二）实施城镇下岗人员再就业工程。落实各项再就业优惠政策，帮扶3800名城镇下岗人员实现再就业。

　　这12件实事，件件体现民意，件件关乎民生，我们一定精心组织，落实到位，把这些实事办成办好、办出实效，让人民群众共享改革发展成果。

　　十、坚持统筹兼顾，扎实推进社会各项事业全面发展

　　大力发展科教事业　开展第四轮创新计划活动，积极推广先进实用技术，深化科技特派员制度，加大科技支农力度。加强高新技术成果的引进，促进科技成果转化，扶持发展高新技术产业。加强知识产权保护和科普推广工作，不断提高全民科技文化素质。抓好梧州职业学院、岑溪职业学校等一批教育项目建设，打好中等职业教育攻坚战，进一步优化教育结构，落实教育惠民政策，切实保障农村义务教育经费，健全贫困学生资助制度，巩固和发展“两基”成果，全面提高义务教育发展水平，促进城乡义务教育均衡发展。

　　加快发展文化事业　把构建社会主义核心价值体系融入文化事业发展中，加快文化体制改革，促进文艺精品创作。以龙母歌舞剧打造为着力点，推动龙母文化产业发展。以“璀璨广场”和“和谐文化进社区”为龙头，丰富城乡社会文化生活。以第三次文物普查为抓手，加强文化遗产发掘保护。以市场管理为重心，促进文化市场健康发展。大力发展公益性文化事业，重点抓好一批公益性项目建设，保障人民基本文化权益。

　　积极发展卫生、体育等社会事业　进一步加大卫生投入，强化公共卫生管理职能，加强公共卫生和医疗服务体系建设，加快市疾控中心实验楼、市医疗紧急救援指挥中心等卫生基础设施建设，完善社区卫生服务中心和乡镇卫生院功能，提高整体医疗服务水平。大力发展体育事业，广泛开展群众体育活动，办好市第十届运动会，提高竞技体育水平。积极发展新闻出版和广播电视事业，坚持正确导向，培育文明风尚。进一步完善和落实人口和计划生育目标管理责任制，健全人口和计划生育工作新机制。加大综合治理出生人口性别比力度，加强流动人口计生管理和服务，严肃查处违法生育行为，强化社会抚养费的征收工作。深入实施人才强市战略，加强各级各类人才队伍建设，制定优秀专家、专门人才激励机制，鼓励扶持他们勇于创业。进一步加强国防和民兵预备役建设，提高民兵预备役执行任务的能力。深入开展拥军优属活动，巩固和发展军政军民团结。全面落实侨务政策，依法保护归侨、侨眷合法权益。加快档案信息化建设。完善社会专项事务管理，发展慈善事业。抓好民族宗教工作，扎实做好外事、对台、气象、防震减灾、修志、测绘以及工会、共青团、妇女儿童、残疾人、老龄等工作，促进社会事业全面发展。

　　加强精神文明和民主法制建设　以落实《公民道德建设实施纲要》为主线，以创建自治区第五轮“文明城市”为龙头，扎实推进群众性精神文明创建活动，切实提高市民素质和城市文明程度。进一步加强公民思想道德、未成年人思想道德教育。以政务诚信、商务诚信、社会诚信为重点，推进社会信用体系建设。深入开展“五五”普法活动，提高全民法制意识。加大纠风治乱工作力度，切实维护群众利益。进一步加强城市社区和农村基层政权建设，适当提高农村基层干部生活待遇。

　　全力维护社会稳定和加强安全生产　深入开展“平安梧州”创建活动，依法防范和打击违法犯罪活动，保障人民生命财产安全。加强社会治安综合治理，完善社会利益协调机制和矛盾纠纷调处机制，做好社会矛盾纠纷排查调处，把各种矛盾和问题解决在基层和萌芽状态。认真落实安全生产责任制，加强安全生产监督和管理，深入开展重大隐患排查和整治，坚决遏制重特大事故发生。加强产品质量和食品药品安全管理，确保食品药品安全。

　　各位代表，新形势、新任务对各级政府提出了新的更高要求，我们必须加强政府自身改革和建设，着力建设服务型政府。我们将在新一轮思想大解放中转变思想观念、转变政府职能，着重提高社会管理和公共服务能力，着力改善民生问题。坚持依法行政，严格按照法定权限和程序履行职责，努力提高政府执行力和公信力。深入落实行政许可法，减少行政审批事项，规范审批和许可行为。自觉接受人民代表大会及其常委会的依法监督，接受人民政协的民主监督，不断提高办理人大代表议案、建议和政协委员提案质量。认真听取民主党派、无党派人士及各人民团体意见，接受新闻媒体的舆论监督和公众的社会监督，不断改进政府工作。加快实行政府绩效管理和行政问责制度。认真落实党风廉政建设责任制，严肃查处违法违纪案件和腐败分子，进一步推进廉政建设。深入贯彻实施《公务员法》，加强对公务员的教育、管理和监督。完善公共服务体系，推进电子政务建设，大力推进政务公开。深入推进作风效能建设，提高工作效率，降低行政成本，完善政务服务中心工作机制，提高为民服务水平。

　　各位代表，我们站在新的历史起点上，新的形势催人奋进，新的目标激发豪情。让我们高举中国特色社会主义伟大旗帜，全面贯彻落实党的十七大精神，在市委的正确领导下，紧紧依靠和带领全市人民，解放思想，抢抓机遇，同心同德，锐意进取，努力谱写梧州人民美好生活新篇章，以优异的成绩向自治区成立50周年大庆献礼！
